# Molecular Mechanisms Regulating Muscle Plasticity in Fish

**DOI:** 10.3390/ani11010061

**Published:** 2020-12-30

**Authors:** Prasanthi Koganti, Jianbo Yao, Beth M. Cleveland

**Affiliations:** 1 Division of Animal and Nutritional Sciences, West Virginia University, Morgantown, WV 26506-6108, USA; ppk26@cornell.edu (P.K.); Jianbo.Yao@mail.wvu.edu (J.Y.); 2 USDA ARS National Center for Cool and Cold Water Aquaculture, Kearneysville, WV 25430, USA

**Keywords:** fish, miRNA, epigenetics, growth, muscle, DNA methylation, muscle, plasticity

## Abstract

**Simple Summary:**

Muscle plasticity is defined as the ability of the muscle to respond to changes in environmental conditions. Muscle plasticity is exceptionally dynamic in fish; this is attributed in part to their ectothermic (cold-blooded) nature and ability of indeterminate or continual growth, throughout their lifespans. The molecular mechanisms regulating muscle growth in fish are not completely characterized; however, recent advancements have established that microRNAs and DNA methylation are important mechanisms regulating muscle plasticity. This review examines these mechanisms and describes how they are regulated by genetic and environmental (i.e., nutrition, temperature) factors and they in turn affect muscle growth and plasticity in fish.

**Abstract:**

Growth rates in fish are largely dependent on genetic and environmental factors, of which the latter can be highly variable throughout development. For this reason, muscle growth in fish is particularly dynamic as muscle structure and function can be altered by environmental conditions, a concept referred to as muscle plasticity. Myogenic regulatory factors (MRFs) like Myogenin, MyoD, and Pax7 control the myogenic mechanisms regulating quiescent muscle cell maintenance, proliferation, and differentiation, critical processes central for muscle plasticity. This review focuses on recent advancements in molecular mechanisms involving microRNAs (miRNAs) and DNA methylation that regulate the expression and activity of MRFs in fish. Findings provide overwhelming support that these mechanisms are significant regulators of muscle plasticity, particularly in response to environmental factors like temperature and nutritional challenges. Genetic variation in DNA methylation and miRNA expression also correlate with variation in body weight and growth, suggesting that genetic markers related to these mechanisms may be useful for genomic selection strategies. Collectively, this knowledge improves the understanding of mechanisms regulating muscle plasticity and can contribute to the development of husbandry and breeding strategies that improve growth performance and the ability of the fish to respond to environmental challenges.

## 1. Introduction

Fish species that exhibit indeterminate growth display an exceptional ability for continual muscle growth throughout their life by an increase in muscle fiber number (hyperplasty) and in muscle fiber size (hypertrophy) [1,2,3,4,5,6,7]. In fish, myogenesis starts during embryonic development by somites from the mesodermal layer. Somites differentiate to myogenic precursor cells (MPCs) that are stored as a reservoir between the basal lamina and sarcolemma of mature muscle bundles and are sequestered during adult myogenesis [8]. Myogenesis is a complex mechanism initiated by activation, proliferation, differentiation, and maturation of MPCs; these processes are orchestrated by various myogenic regulatory factors (MRFs), signaling pathways, non-coding RNAs, and epigenetic mechanisms. The functional aspects of MRFs and signaling pathways in mammalian species are well documented in previous reviews [9,10,11,12], hence their role in skeletal muscle development is briefly discussed in the current review. Our focus is to discuss the role of epigenetic and microRNA mechanisms in myogenesis and their regulation by biological factors thus affecting muscle plasticity in fish.

## 2. Myogenic Regulatory Factors

Myogenic regulatory factors are basic helix-loop-helix transcription factors that drive the process of myogenesis, including MPC proliferation and differentiation (Table 1). Preferential expression of MRFs determines progression of MPCs [13]. It is accepted that MRF function is generally conserved in animals; this concept is supported by MRF protein sequences with high similarity and consistent expression patterns throughout MPC progression. Myf5 is an important MRF that functions to promote the commitment of satellite cells to myogenic lineage. The committed MPCs are cells that express MRF specific to satellite cells, including Pax7 [14,15], HGF receptor c-met [15], and Syndecan-4 [16]. Pax7 is necessary for the maintenance of satellite cells state [8,17,18,19,20]. Absence of Pax7 expression leads to defective muscle differentiation through cell cycle arrest and precocious differentiation [21,22]. MPCs enter a proliferative phase and are capable of asymmetric and symmetric cell division to either self-renew or differentiate into myoblasts. The self-renewed cells express high levels of Pax7 [18] and those that differentiate express high MyoD with a decrease in Pax7 expression [23]. Thus, MyoD is one of the major factors determining the myogenic cell fate of MPCs [24,25]. In addition to the MRFs necessary for satellite cell maintenance and differentiation, the expression of Mrf4 and Myogenin after proliferation and during differentiation marks the differentiation process to form a myofiber [26,27].

Signaling molecules play a significant role in regulating the expression of these MRFs, thus determining the fate of myogenesis. TGF-β family members are responsible for satellite cell maintenance while hedgehog signaling is responsible for transition from proliferative to quantile mitosis. Satellite cell markers Pax7 and Pax3 are downregulated and differentiation factor MyoD is upregulated by hedgehog in teleosts, thus determining myogenic commitment. Additionally, insulin-like growth factor-I (IGF-I) and insulin regulate myogenesis by binding to receptor tyrosine kinase and initiating signaling cascades. Growth factor signaling enhances myogenic proliferation and differentiation via mitogen-activated protein kinase (MAPK), Ras/Raf, and PI3K pathways thus controlling muscle regeneration, MPCs proliferation, and differentiation, respectively [34]. The signaling pathways initiate a cascade of reactions by phosphorylating different intermediates, including target of rapamycin (TOR), thereby activating MRFs and ultimately triggering gene expression responsible for muscle synthesis [35].

## 3. Epigenetics and DNA Methylation

Chemical modification of DNA bases was first reported in *Tubercle bacillus* and later in calf thymus DNA [36,37]. Further establishing of the importance of DNA methylation in transcriptional regulation of eukaryotic gene expression was reported by two simultaneous studies [38,39]. Since then, studies have reported the presence of such modifications in various organisms including prokaryotes, fungi, plants, and animals, including fish. These modifications are post-synthetic, as the methyl group is added after the nucleotides are incorporated into the DNA. Methylation of bases is carried out by DNA methyltransferases (Dnmt) and the methyl group is donated by S-adenosyl methionine. DNA methylation in mammals is carried out by three Dnmts: Dnmt1, Dnmt3a, and Dnmt3b. However, eight Dnmt genes were identified in zebrafish and included Dnmt1 and Dnmt2, while the rest were similar to the mammalian Dnmt3 [40,41,42]. Dnmt1 is a maintenance methyltransferase mainly functioning in methylating hemi-methylated DNA strands during replication. Dnmt3a and Dnmt3b are de novo methyltransferases methylating nascent DNA or hemi-methylated DNA. De novo methyltransferases are essential for laying methylation marks during embryo implantation and early development [43]. The majority of base modifications in eukaryotes are on cytosines, while some of the unicellular organisms present methylation on adenosine [44,45]. A methyl group is added to the carbon at fifth position of cytosine resulting in 5^m^C. Until recently it was believed that methylation of cytosine is restricted to CpG dinucleotide. Advanced sequencing techniques as well as the ability to map sequences at base pair resolution disclosed non-CpG methylations. Non-CpG methylations are less frequent than CpG methylations and are seen in mammalian oocytes [46], adult brain [47], and embryonic stem cells [48]. In general, DNA methylation is associated with transcriptional silencing, although there are exceptions [49]. Introduction of the methyl group on cytosine affects the binding of proteins such as repressors, histones, and hormone receptors to the DNA indicating regulation of gene expression [50,51,52] by sterically preventing binding. The methylated regions act as binding sites for certain transcriptional repressors including MeCP2, Mbd1, and Mbd2 leading to closed chromatin [53].

### 3.1. DNA Methylation during Skeletal Myogenesis

Studies in mammals confirmed the regulatory role of DNA methylation during myogenesis, including activation of MPC, proliferation, and differentiation. Comparative studies to understand the methylation landscape among myogenic differentiating cells and mature skeletal muscle suggested loss of methylation in mature fibers. In mice differentiating muscle cells exhibit approximately 90% higher methylation rates when compared to mature myofibers [29]. Differential expression of enzymes involved in DNA methylation was observed in a stage specific manner; Dnmt1 was upregulated during activation and downregulated during differentiation. Reduced expression of de novo methyltransferases Dnmt3a and demethylating enzymes Tet1, Tet2, and Tet3 during muscle precursor cell activation was reported while Dnmt3b remained unchanged [54,55,56,57]. The role of DNA methylation in cell fate commitment and in progression of myogenesis was supported by several mammalian studies using 5-azacytidine and antisense RNA to inhibit Dnmt1 and DNA methylation [58,59]. Proliferating myoblasts exposed to 5-azacytidine exhibited increased expression of myogenic genes including Myogenin. This indicated a functional role of DNA methylation in permitting binding of myogenic transcription factors to their target genes promoting differentiation [60]. Hyper and hypomethylation of various MRFs and genes involved in the process of myogenesis are listed in Table 1. Collectively these studies support the important role of DNA methylation in various stages on myogenesis. Although most of the studies represent CpG methylations, the importance of non-CpG methylations is yet to be identified. Additionally, technical methods used could not differentiate between methylated and hemi-methylated cytosines, hence studies understanding such differences are necessary to know the functional impact of DNA methylation in myogenesis.

### 3.2. Epigenetic Regulation of Muscle Plasticity in Fish

Most advances in the knowledge of epigenetic regulation of muscle plasticity in fish have occurred only within the last five years; findings have provided support for DNA methylation as a mechanism affecting muscle growth (Table 2). Comparing the methylome of fast and comparatively slow growing tilapia indicated approximately 1000 differentially methylated CpGs in both males and females, although there was very little overlap between the sexes [61]. These findings indicate that variations in DNA methylations are associated with faster growth, providing strong support for epigenetic mechanisms as a significant regulator of muscle plasticity. In particular, the autophagy-related gene Atg14 displayed a high association of methylation with growth in male tilapia, suggesting that suppression of muscle protein degradation contributes to muscle growth. Research in zebrafish reports similar findings; in muscle the autophagy-related genes Atg4b and Lc3b are both hypomethylated and up-regulated during nutrient deprivation [62], supporting that DNA methylation is responsible for starvation-induced loss of muscle protein. Nutritional regulation of muscle growth via epigenetic modifications is supported by additional studies in rainbow trout indicating hypermethylation in muscle of fish consuming higher dietary carbohydrates [63] and regulation of DNA methyltransferase expression by dietary protein in the Senegalese sole [64]. This concept is further supported by a nutritional programming study in tilapia in which injections of glucose into the yolk of alevins were associated with DNA hypomethylation in muscle 20 weeks post-injection [65], suggesting the existence of epigenetic mechanisms at the origin of programming that affect muscle growth.

Several studies have characterized the role of DNA methylation in response to temperature variation during early rearing stages. In Atlantic salmon, higher embryonic incubation temperature (4 °C vs. 8 °C) increases post-embryonic growth rates; in first-feeding larvae this response was associated with both reduced DNA methylation of the Myogenin promoter and higher Myogenin expression [66]. Effects of incubation temperature on muscle Dnmt expression extended to the parr stage in which expression of Dnmt1 and Dnmt3a increased with higher larval rearing temperature. Similarly, European sea bass larvae incubated at higher temperatures exhibited growth benefits, differential genomic methylation patterns, and increased expression of Myogenin, Dnmt1, and Dnmt3 [67]. Comparable findings were observed in the Senegalese sole; higher early rearing temperature enhanced hyperplastic muscle growth during metamorphosis [68], a response that was correlated with reduced methylation of the Myogenin promoter, increased Myogenin expression, and regulation of Dnmt1 and Dnmt3a expression [69]. In Atlantic cod, exposure to continuous illumination during early juvenile development improved growth potential and was associated with higher Dnmt1 and Dnmt3a expression in fast muscle. [70]. Although the directional regulation of Dnmt expression is not consistently associated with the faster growth phenotype, these studies provide evidence that temporal effects of early rearing conditions on muscle growth are regulated in part through modification of DNA methylation.

Although most studies have focused on changes in muscle Dnmt expression as an indicator of regulation of DNA methylation capacity, fewer have investigated changes in methylation of MRFs that affect myogenesis and muscle plasticity. As previously mentioned, in two studies improved growth rates induced by higher embryonic incubation temperatures corresponded to hypomethylation of the Myogenin promotor and increased expression in muscle [66,67]. In rainbow trout 17β-estradiol increased methylation within exon 1 of Myod and decreased expression of the Myod gene, providing evidence for down-regulation of a MRF by a steroid treatment that also promotes muscle atrophy [71]. Also in rainbow trout, unique histone methylation patterns of the three paralogous Pax7 genes were detected during in vitro myogenesis [74]. Expression of Pax7a2 exhibited decreased expression with decreased H3K27 trimethylation. In contrast, Pax7b expression increased and was correlated with decreased H3K9me3 and H3K27me3.

## 4. MicroRNA Regulation of Myogenesis

Small RNAs, particularly microRNAs (miRNAs), are extensively studied as post-transcriptional microregulators of gene expression governing either mRNA stability, rates of translation, or both [75]. Studies in mammals provide evidence for their transport [76] and nuclear existence [77], suggesting functions other than translational inhibition. Similarly, the presence of miRNA in exosomes [78] advocates their secretion and circulation through body fluids [79,80,81] to control gene expression in recipient cells [82]. Besides continuous efforts to understand various functional roles of miRNA, their biogenesis is well established [83]. Primary transcripts of miRNA are either generated by transcription of individual miRNA genes or by processing of intronic regions. MicroRNAs bind to target mRNA by either perfect or imperfect base-pairing, leading to translational repression or mRNA degradation. In addition, nuclear miRNAs bind to promoters and either activate or inhibit gene expression. The distinguishable characteristics or functional properties of activating and inhibiting miRNAs are not well understood [83].

### 4.1. Functional Regulation of miRNA in Muscle

Myogenesis involves orchestrated interaction of various mechanisms involving signaling pathways and coordinated gene expression of various myogenic regulatory factors (MRFs). The role of miRNA in myogenesis was first established in Dicer mutant mice exhibiting reduced muscle miRNA, perinatal death, and decreased skeletal muscle with abnormal myofiber morphology and apoptosis of myoblasts [84]. This study established the important role of miRNAs in vertebrate muscle development. MicroRNAs with muscle specific expression and function are designated as myomiRNAs. The involvement of myomiRNAs in MPC proliferation and differentiation is established [85,86,87]. For example, miR-1 and miR-206 promote myoblast differentiation through anti-proliferative effects by repressing expression of the MRFs Pax3, Pax7, and Notch 1. The myomiRNA miR-133 also suppresses proliferation and promotes differentiation through various mechanisms, including repression of the mitogen-activated protein kinase pathway, cyclin D2, and uncoupling protein-2. Nachtigall et al. [88] compared the evolution and organization of myomiRNAs in cartilaginous and bony-fish genomes through genome-wide comparative analysis and concluded synteny in myomiRNA distribution.

### 4.2. MicroRNAs Targeting Genes Involved in Muscle Development

In several fish species, including rainbow trout [89,90], zebrafish [91], Japanese flounder [92], sea bass [93], Nile tilapia [94], Chinese perch [95], and common carp [96], the most abundant miRNAs expressed in skeletal muscle are reported as miR-1, miR-206, and miR-133a. In vivo and in vitro studies in mammalian systems have established that interaction of miR-1, miR-206, and miR-133a with their target genes regulates skeletal muscle cell proliferation and differentiation [97,98,99]. These studies shed light on the regulatory network among the miRNA and MRFs. Mature miR-1 and miR-133 are derived from one polycistronic pre-miRNA targeting two different genes. miR-1 promotes myogenesis by targeting histone deacetylase 4, a transcriptional repressor of muscle gene expression, while miR-133 targets serum response factor which enhances muscle cell proliferation [97]. In zebrafish, miR-1 and miR-133 explain more than 54% of miRNA-induced regulation of gene expression, that when down-regulated cause disruption of actin organization in sarcomere assembly [91]. Additionally, muscle specific expression of miR-206 is controlled by Myod [99] which along with miR-1 targets and represses pax7 that is a marker for proliferation [98]. Regulatory expression of miR-206 by Myod is also associated with the negative regulation of Follistatin-like 1 and Utrophin genes which are necessary for muscle cell differentiation [99]. MicroRNA-206 is also involved in negative regulation of Igf-1 expression in tilapia skeletal muscle [100]. In addition, findings in tilapia and Chinese perch report negative regulation of Myod expression by miR-203b and miR-143, respectively; both miR-203b and miR-143 bind to the 3′-untranslated region (UTR) of MyoD, suppressing its expression [101,102]. Further promoting growth is miR-181a-5p that represses expression of Myostatin-b in tilapia [103]. Repression of Myostatin expression by miR-2014 and miR-1231-5p is also described in yellow croaker (*Larimichthys crocea*) [104].

Unique miRNA expression profiles are reported in muscle during different developmental stages in common carp [96], the ray-finned *Schizothorax prenanti* [105], and pacu (*Piaractus mesopotamicus*) [106], supporting a significant role in maintenance and growth of this tissue. Genetic and environmental factors are also established as significant regulators of miRNA in fish muscle (Table 3). Differences in expression of miRNAs between fish with divergent growth phenotypes suggest that genetic variation in miRNA expression is a significant mechanism affecting the capacity for muscle growth. These findings were reported in rainbow trout [107], tilapia [94], Chinese perch [108], and blunt snout sea bream [109]. Notable was an up-regulation of miR-133 in fast-growing tilapia [94]. Expression of miR-133, miR-181a-5p, and miR-206 is also correlated with body weight in rainbow trout, although indirectly [107]. However, miR-1, miR-133, and miR-206 were down-regulated in fast-growing, compared to slow-growing, Chinese perch [108]. Variation in miRNA expression that correlates with body weight warrants additional research that investigates whether genetic variation in miRNA expression has value as genomic markers for growth phenotypes.

### 4.3. MicroRNA Regulation of Muscle Cell Fate

MicroRNAs can determine muscle cell fate very early during somatogenesis. Fish exhibit two distinct muscle types, slow and fast muscles, and the role of miRNAs in muscle cell type determination was reported by studies using fish as model species. Ubiquitously expressing miR-214 synchronizes Hedgehog signaling in zebrafish by translational repression of its negative regulator Su(Fu), thus coordinating a balance among slow and fast muscle cell types. Knockdown of miR-214 in zebrafish embryos led to the development of fewer to a complete absence of slow muscle cells [120], and hence established miRNA as necessary for development of slow muscle cells. Similarly, knockdown and overexpression of miR-3906 in zebrafish embryos affected the fast muscle phenotype [121] through its function in maintenance of calcium ion concentration homeostasis specifically in fast muscles. Knockdown of miR-3906 increases the expression of its target gene Homer-1b, which in turn up-regulates fast muscle specific gene Fmhc4 and calcium sensitive gene Atp2a1, thus causing a surge in calcium ions concentration and disorganized sarcomeric actin in fast muscle resulting in swimming abnormality. However, over expression of miR-3906 decreases calcium ion concentration, resulting in bent bodies and shortened tails in zebrafish [121]. Another important miRNA extensively studied for its involvement in muscle cell fate determination is miR-499; it is highly expressed in slow skeletal muscle in Nile tilapia, pacu, and rainbow trout compared to fast skeletal muscle [88,106,122]. A regulatory network involving functional repression of Sox6 by miR-499 through Prdm1 for the maintenance of slow-twitch muscle was also reported in zebrafish [123], allowing restricted expression of Sox6 in fast-twitch muscle. In the Chinese perch, miRNA profiling in fast and slow muscle suggests that miR-181a-5p, miR-143, and miR-103 have central roles in regulating the performance of the muscle types [95,102,124]. All together these studies emphasize the regulatory mechanisms of miRNA in balanced determination of muscle cell fate and muscle-specific function.

### 4.4. Biological Factors Affecting miRNA Expression in Muscle

Understanding the role of miRNAs as a mechanism regulating muscle function during physiological perturbation has been the focus of numerous studies in fish. Perhaps one of the most significant environmental factors affecting muscle growth in fish is the availability of nutrients; several studies support that both nutrient intake and diet composition affect miRNA expression in muscle. Experimental designs that involve feed deprivation and refeeding have been valuable for identifying miRNA biomarkers for anabolic and catabolic responses. When Chinese perch [112] and grass carp [113] are subjected to feed deprivation and refeeding, miR-206, miR-133a-3p, and miR-181a-5p are up-regulated within just 1–3 h of refeeding, supporting that miRNA-induced regulation of MRFs like Myod and Myostatin are significant for rapid recovery growth. Additional in vivo and in vitro studies have investigated effects of specific nutrients on miRNA expression using rainbow trout and MPCs derived from its muscle. Methionine deficiency arrested cell differentiation and reduced expression of miRNAs, including miR-133a and the MRFs Myod and Myogenin, which were rescued by the introduction of methionine [114]. Analogous to in vitro experiments, decreased expression of miR-133a was also observed in muscle of rainbow trout consuming a methionine-deficient diet [115]. In tilapia muscle it was determined that regulation of Kruppel-like factor-15 (Klf15), a key regulator of branched-chain amino acid metabolism, during feed deprivation is mediated by changes in miR-125a-3p expression [116]. This mechanism is likely significant for the metabolic and physiological adaptations to nutrient deprivation in fish. Furthermore, miRNAs with established roles as regulators of myogenesis (i.e., miR-1, miR-133, miR-206) are affected by consumption of first feeds in the rainbow trout alevin [117] and Atlantic cod [118].

Temperature is an additional factor that affects miRNA expression. In zebrafish, changes in muscle growth trajectories (hyperplasia vs. hypertrophy) induced by manipulation of embryonic incubation temperature were associated with differential expression of miRNAs, with increased Myogenin expression in the higher temperatures that improved growth performance [110]. Similar findings are also reported in Senegalese sole, in which thermal plasticity of muscle growth was correlated with increased Myogenin expression and regulation of miRNAs with putative roles in muscle development and nutrient metabolism, such as miR-181-5p/3p and miR-206-3p [111,125]. These studies point towards miRNA-related mechanisms that regulate muscle plasticity during temperature manipulation and fluctuation; this information can contribute to the optimization of husbandry strategies to enhance muscle growth or improve fish health and performance. The need for such studies to understand the consequences of climate change on fish species is increasing.

In addition to biological factors of exogenous origin, endogenous factors such as hormones are established as regulators of muscle physiology through mechanisms involving miRNAs. Physiological changes in salmonid skeletal muscle during sexual maturation are characterized by protein degradation and lipid loss [126,127]. By utilizing the spawning salmonid as a model for muscle atrophy, studies in rainbow trout have determined that miRNAs are important for the mobilization of muscle nutrient reserves during the energy intensive spawning period. Through comparing the transcriptome of sterile and spawning rainbow trout, 28 miRNAs were associated with the lncRNA-mRNA-microRNA gene network described as the muscle ”degradome” [119]. The steroid hormone, 17β-estradiol, is up-regulated only during sexual maturation in female rainbow trout and has been implicated as a biological factor directly regulating miRNA in muscle [90]. Furthermore, 17β-estradiol-induced responses in muscle included regulation of miR-133, miR-206, and miR-499, along with differential regulation of MRFs like Pax7 and Myod. In an additional area of hormone-related research, efforts to understand the effects of thyroid hormone influence on histone deacetylase (Hdac4) lead to the discovery of miRNA regulation of muscle development during metamorphosis in the Japanese halibut [105]. This study presented evidence of an interaction between signaling pathways, epigenetic regulators like Hdac4, and miRNAs (miR-1 and miR-133).

## 5. Conclusions

Within the last decade significant advancements in the knowledge of molecular regulation of gene expression have established that miRNAs and DNA methylation are significant mechanisms affecting expression of myogenic regulatory factors and muscle plasticity in fish. Genetic, environmental, and physiological factors cause differential expression of miRNAs and DNA/gene methylation in muscle that are directly linked or correlated with growth responses. These findings are reported in model species like the zebrafish and stickleback as well as fish species significant for aquaculture such as rainbow trout, Atlantic salmon, Chinese perch, and tilapia. Continued advancements in our understanding of the molecular mechanisms regulating muscle plasticity is central for the development of novel genetic markers to aid in selective breeding for enhanced growth. It is also valuable for the development of husbandry strategies that improve the capacity for muscle growth through, for example, diet, photoperiod, or temperature manipulation that enhance aquaculture production efficiency and advance global food security.

## Figures and Tables

**Table 1:** Established roles of DNA methylation for myogenic regulatory factors (MRF).

Gene/MRF	Functional Role	Methylation Status	Reference
Pax7	Migration and early lineage commitment	Hypermethylation in myogenic cells and mature muscle fibers	[28]
Myf5	Proliferation and differentiation of MPC into myoblasts	Hypermethylation of enhancer region in embryonic stem cells Hypomethylation in myoblasts, myotubes, and skeletal muscle	[29]
Myod	Proliferation and differentiation of MPC into myoblasts	Hypomethylation in distal enhancer region	[30]
Myogenin	Differentiation of myoblasts into myotubes	Demethylation in differentiated muscle Hypermethylated in myoblasts and non-myogenic cells	[31]
Obsn	Formation of skeletal muscle	Hypomethylation in muscle tissue Hypermethylated in myoblasts and myotubes	[28]
Myh7b	Expressed intronic microRNA miR499	Hypomethylation	[28]
Gene promoters	Myotube formation	Hypermethylation of ID4 and ZNF238 binding sites	[32]
Notch1	Proliferation of muscle satellite cells	Hypomethylation and its ligands Dll1 and Jag2 in skeletal lineage cells	[33]

**Table 2:** Research in fish investigating epigenetic regulation of muscle plasticity.

Factor Affecting Muscle Plasticity	Fish Specie(s)	Reference
Genetic variation in growth	Nile tilapia	[61]
Temperature	Atlantic salmon, European sea bass, Senegalese sole, stickleback	[66,67,68,69]
Nutrition	zebrafish, rainbow trout, Senegalese sole	[62,63,64,65]
Photoperiod	Atlantic cod	[70]
17β-estradiol	rainbow trout	[71]
Seasonal acclimation	common carp gilthead sea bream	[72,73]

**Table 3:** Research in fish supporting miRNA-mediated regulation of muscle plasticity.

Biological Factors Affecting Muscle Plasticity	Fish Specie(s)	Reference
Temperature	Senegalese sole zebrafish	[110,111]
Genetic variation in growth	rainbow trout tilapia Chinese perch, blunt snout sea bream	[94,107,108,109]
Nutrition	rainbow trout, Chinese perch, grass carp, Nile tilapia, Atlantic cod	[112,113,114,115,116,117,118]
Spawning or 17β-estradiol	rainbow trout	[90,119]

## Data Availability

No new data were created or analyzed in this study. Data sharing is not applicable to this article.
